# First Record of Microbiomes of Sponges Collected From the Persian Gulf, Using Tag Pyrosequencing

**DOI:** 10.3389/fmicb.2018.01500

**Published:** 2018-07-06

**Authors:** Akram Najafi, Maryam Moradinasab, Iraj Nabipour

**Affiliations:** ^1^The Persian Gulf Marine Biotechnology Research Center, The Persian Gulf Biomedical Sciences Research Institute, Bushehr University of Medical Sciences, Bushehr, Iran; ^2^The Persian Gulf Tropical Medicine Research Center, The Persian Gulf Biomedical Sciences Research Institute, Bushehr University of Medical Sciences, Bushehr, Iran

**Keywords:** sponge, symbionts, bacterial diversity, 454 pyrosequencing, the Persian Gulf

## Abstract

The Persian Gulf is a special habitat of marine sponges whose bacterial communities are under-investigated. Recently, next-generation sequencing technology has comprehensively improved the knowledge of marine sponge-associated bacteria. For the first time, this study aimed to evaluate the diversity of the Persian Gulf sponge-associated bacteria using tag pyrosequencing in Iran. In this study, 10 sponge samples from 6 different taxonomic orders were collected from the Persian Gulf using SCUBA diving. The diversity of the bacteria associated with the marine sponges was investigated using the *16S rRNA* gene PCR-tagged pyrosequencing method. A total of 68,628 high-quality sequences were obtained and clustered at a 97% similarity into 724 unique operational taxonomic units (OTUs), representing 17 bacterial phyla. Cyanobacteria was the most abundant phylum in the sponges, followed by Proteobacteria, Chloroflexi, Acidobacteria, and Actinobacteria. Other phyla were detected as minor groups of bacteria. Bacterial community richness, Shannon, and Simpson indices revealed the highest diversity in sponge S11 (*Dictyoceratida* sp.) compared to other sponges. This study showed a diverse structure of bacterial communities associated with the Persian Gulf sponges. The dominance of Cyanobacteria may suggest an ecological importance of this phylum in the Persian Gulf sponges.

## Introduction

Marine sponges (phylum *Porifera*) are known as the oldest multicellular animals (metazoans) (found more than 600 million years ago) (Lee et al., 2011; Verhoeven et al., 2017) and represent ecologically important reef builders in benthic communities' worldwide (Bayer et al., 2014; Graça et al., 2015). In the last decade, sponges have attracted research interests because of their symbiotic relationships with a wide range of microbial communities including bacteria (Lee et al., 2011; Giles et al., 2013; Gao et al., 2014a), archaea (Zhang et al., 2014), Cyanobacteria (Gao et al., 2017; Regueiras et al., 2017), and fungi (Maldonado et al., 2005). The sponge-associated microorganisms can constitute up to 40–60% of the total sponge biomass (Gao et al., 2014a; Graça et al., 2015; Gaikwad et al., 2016). They may play crucial roles in sponge survival in the marine ecosystem including recycling of nutrients such as nitrogen and sulfur (Montalvo et al., 2014; Zhang et al., 2014), removing metabolic waste (Jackson et al., 2012), and producing bioactive secondary metabolites (Graça et al., 2015).

Extensive studies have been conducted to investigate the bacterial communities associated with different sponge species using both culture-dependent and culture-independent techniques (Giles et al., 2013; Jeong et al., 2014). As is common with most environments, < 1% of bacteria present in sponge tissues can be successfully cultivated (Nam et al., 2011; Jackson et al., 2012). In the past two decades, culture-independent methods (mainly based on *16S rRNA* gene) have led to a deeper understanding of the microbial diversity in sponges (Alex and Antunes, 2015). Numerous sponge-associated bacteria have been identified using culture-independent techniques such as denaturing gradient gel electrophoresis (DGGE) (Li et al., 2006), fluorescent *in situ* hybridization (FISH) (Bayer et al., 2014), terminal restriction fragment length polymorphism (TRFLP) analyses (Zhang et al., 2006; Lee et al., 2011), PCR cloning and sequencing (Kennedy et al., 2008). The 454 tag pyrosequencing is a next-generation sequencing (NGS) technology that provides a faster and simpler way to analyze the microbial communities associated with marine sponges (Webster et al., 2010; Lee et al., 2011; Schmitt et al., 2012; Gao et al., 2014a; Gaikwad et al., 2016). This new method enables hundreds of thousands of nucleotide sequences from multiple samples to be examined in a single 10 h reaction (Lee et al., 2011; Nam et al., 2011; Jeong et al., 2013, 2014).

The next-generation sequencing techniques have revealed the presence of more than 25 different bacterial phyla and 2 archaeal lineages in marine sponges around the world (Moitinho-Silva et al., 2014; Rodríguez-Marconi et al., 2015; Verhoeven et al., 2017). Members of the phyla Actinobacteria, Acidobacteria, Cyanobacteria, Chloroflexi, Proteobacteria, Bacteroidetes, and Firmicutes have been described in association with different marine sponges (Jackson et al., 2012; Giles et al., 2013; Bayer et al., 2014). However, the marine bacterial communities can vary in different sponges with respect to both microbial richness and diversity.

The Persian Gulf, a small, shallow, semi-enclosed body of water bordered by the Arabian Peninsula and Iran, is a unique and greatly underexplored marine ecosystem. There are about 55 sponge genera recorded in the Persian Gulf (Najafi, 2012). However, next-generation sequencing technology has not been applied to identify the sponge-associated bacterial communities in Iran and the Middle East.

The present study was aimed at characterizing the bacterial community associated with the marine sponge species collected from the Persian Gulf, Iran using 454 pyrosequencing.

## Materials and methods

### Sponge sampling

Sponge sampling was performed in May to September 2016 at a depth of 2–3 m offshore Bushehr, Persian Gulf, Iran by SCUBA diving. In this study, the sponges were living in an area where it was exposed to light. Sponge samples were placed in sterile plastic Ziploc bags containing seawater and immediately transported to the Persian Gulf of Marine Biotechnology Research Center. Sponge tissues were rinsed with 0.22-μm-membrane-filtered seawater (FSW) to remove exogenous materials and loosely attached microbes (Jackson et al., 2012). The samples were stored at −80°C until further processing.

### Ethics statement

In this study, the sponges collected did not involve endangered or protected sponge species. No specific scientific research permission was required to collect the sponges from the Persian Gulf.

### Sponge identification

Sponge taxonomic identifications were confirmed with a combination of multilocus DNA markers. The cytochrome oxidase subunit 1 (COI) and partial 28S rDNA fragments (ITS) were amplified using specific primers as previously reported (Becking et al., 2013). PCR amplifications were carried out on a thermal cycler PeQlab, peqSTAR 96X Universal Gradient, Germany under the following conditions: 94°C for 30 s; followed by 35 cycles of 94°C for 5 s; 50°C for 5 s; 72°C for 12 s; followed by 72°C for 1 min (Becking et al., 2013). PCR products were purified and sequenced by Macrogen Inc. (Seoul, Korea).

Also, morphological and spicule examination was carried out by Dr. Yusheng M. Huang (National Penghu University of Science and Technology, Taiwan). List of sponge species collected from different locations of the Persian Gulf is shown in Table 1.

**Table 1:** List of sponge species collected from different locations of the Persian Gulf.

**Site**	**Collection date**	**Coordinate**	**Depth (m)**	**Temp (C)**	**Sample ID**	**Sponge species**	**Taxonomy**		
							**Class**	**Order**	**Family**
PG1	September 2016	27.8238 N, 51.8948 E	~2	32.0	S02	*Suberites diversicolor*	*Demospongiae*	*Suberitida*	*Suberitidae*
PG1	September 2016	27.8238 N, 51.8948 E	~2	32.0	S03	*Pseudoceratina arabica*	*Demospongiae*	*Verongida*	*Pseudoceratinidae*
PG2	July 2016	28.9817 N, 50.8243 E	3	29.6	S04	*Chondrilla* sp.	*Demospongiae*	*Chondrillida*	*Chondrillidae*
PG2	July 2016	28.9817 N, 50.8243 E	3	29.6	S05	*Cladocroce* sp.	*Demospongiae*	*Haplosclerida*	*Chalinidae*
PG2	July 2016	28.9817 N, 50.8243 E	3	29.6	S06	*Cladocroce* sp.	*Demospongiae*	*Haplosclerida*	*Chalinidae*
PG2	July 2016	28.9817 N, 50.8243 E	3	29.6	S07	*Halichondria* sp.	*Demospongiae*	*Halichondrida*	*Halichondriidae*
PG3	May 2016	28.9816 N, 50.8253 E	3	28.0	S09	*Chondrilla* sp.	*Demospongiae*	*Chondrillida*	*Chondrillidae*
PG3	May 2016	28.9816 N, 50.8253 E	3	28.0	S10	*Halichondria* sp.	*Demospongiae*	*Halichondrida*	*Halichondriidae*
PG3	May 2016	28.9816 N, 50.8253 E	3	28.0	S11	Dictyoceratida sp.	*Demospongiae*	*Dictyoceratida*	Unclassified Dictyoceratida
PG3	May 2016	28.9816 N, 50.8253 E	3	28.0	S13	*Ircinia ramose*	*Demospongiae*	*Dictyoceratida*	*Irciniidae*

### Metagenomic DNA extraction from sponges

Frozen sponge tissues were defrosted and washed with sterilized and filtered seawater. Then, they were cut into small pieces (about 1 cm^3^) and ground to fine powder under liquid N_2_ using a sterile pestle and mortar (Jackson et al., 2012; Bayer et al., 2014). DNA was extracted using a hexadecyltrimethylammonium bromide (CTAB) method. Briefly, a subsample of approximately 100 mg of each sponge tissue was suspended in lysis buffer [100 mM Tris, 100 mM EDTA, 1.5 M NaCl (w/v), 1% CTAB (w/v), 2% SDS (w/v)]. Then they were disrupted in the presence of factors such as proteinase K and extraction buffer containing chloroform: isoamyl alcohol (24:1). Samples were washed with a solution of phenol-chloroform in a few steps for DNA purification. Finally, DNA was precipitated with sodium acetate (3 M, pH 5.2) and isopropanol, then washed in 70% ethanol, dried and re-dissolved in TE buffer (Jackson et al., 2012; Schmitt et al., 2012). Metagenomic DNA was qualified by agarose gel electrophoresis [1% *w/v* agarose in Tris-acetate-EDTA (TAE) buffer]. The quantitative assessment of the isolated DNA was carried out using a NanoDrop 1000 spectrophotometer (Thermo Scientific, Wilmington, DE, USA). The high-quality DNA was stored at −20°C until use (Schmitt et al., 2012; Jasmin et al., 2015).

### Pyrosequencing of barcoded *16S rRNA* gene amplicons

In this study, the universal primers 27F and 518R were used to amplify a ~ 400 bp fragment of the bacterial *16S rRNA* gene targeting the V1 to V3 hyper-variable regions. These regions were amplified using primer sets (V1-27F: 5′-X-MID-GAGTTTGATCMTGGCTCAG-3′ and V3-518R: 5′-X-MID-WTTACCGCGGCTGCTGG-3′), in which X indicates the adapter sequences and MID (multiplex identifier) shows the different oligomers comprised of 10 nucleotides to tag different samples for barcoded pyrosequencing (Table 2; Jeong et al., 2013, 2014). This approach allowed for the mixing of multiple samples in parallel and re-sorting the sequences into order (Nam et al., 2011; Jackson et al., 2012). PCR amplification was performed in a volume of 50 μL containing 3 mM of MgCl_2_, 0.2 mM of dNTPs, 2.5 U of Pfu Turbo DNA polymerase (Stratagene, La Jolla, CA, USA), 1X Pfu reaction buffer, 0.1 μM of each pair of barcoded primers, and 20 ng of metagenomic DNA (Lee et al., 2011; Gao et al., 2014a). PCR was conducted in a Thermal Cycler (Applied Biosystems ABI Perkin Elmer 9600 GeneAmp) using the following conditions: an initial denaturation at 95°C for 5 min, followed by 35 cycles of denaturation at 95°C for 60 s, annealing at 55°C for 60 s, extension at 72°C for 60 s, and a final extension at 72°C for 5 min (White et al., 2012). The PCR amplicon libraries were purified using the NucleoSpin® Gel and PCR Clean-up (Macherey-Nagel, Germany) and quantified using NanoDrop 1000 spectrophotometer (Thermo Scientific, Wilmington, DE, USA). Pyrosequencing was performed through a GS FLX Titanium system (454 Life Sciences) according to the manufacturer's instructions (Roche, Germany) by a commercial sequencing provider (Macrogen, Seoul, Korea).

**Table 2:** The MID barcodes for the amplification of *16S rRNA* genes.

**Sample**	**S2**	**S3**	**S4**	**S5**	**S6**	**S7**	**S9**	**S10**	**S11**	**S13**
MID	ACGAG TGCGT	ACGCT CGACA	CTCTA CGCTC	AGCAC TGTAG	ATCAG ACACG	ATATC GCGAG	CTGTA CATAC	CTCGC GTGTC	TAGAC TGCAC	TGATA CGTCT

### Processing of 454 tag sequences data

In the present study, the sequences generated from pyrosequencing were analyzed as previously reported (Jeong et al., 2014). The low-quality sequences were filtered from the raw reads using Trimmomatic v0.30 (Gaikwad et al., 2016). Briefly, sequences with a read length of less than 172 bp or with mismatches on primer or barcode, a quality score of less than 25 with ambiguous bases N and homopolymers longer than 6 nucleotides were removed from further analysis (Gao et al., 2014a; Moitinho-Silva et al., 2014; Rodríguez-Marconi et al., 2015; Gaikwad et al., 2016). Final sequences that passed the quality checks were then assigned to respective samples based on the barcodes and subjected to Denoiser to increase the accuracy of the sequence processing (Gao et al., 2014a). The sequences were merged into one file and clustered into operational taxonomic units (OTUs) using the Quantitative Insights Into Microbial Ecology Toolkit (QIIME), version 1.8.0. (Caporaso et al., 2010). Chloroplast and mitochondria sequences and chimeric reads were excluded from downstream analyses. In this study, the chimeric reads were removed through the CD-HIT-OTU program (http://cd-hit.org). Taxonomic assignment of representative OTU sequences was performed using the UCLUST (version 1.2.22) taxonomy assigner method (Edgar, 2010) against the SILVA release 119 database as a reference. To have more information about the strains, each OTU was compared to the most closely related *16S rRNA* gene sequences from the NCBI nucleotide databases using BLAST search. Phylogenetic analysis was inferred by using the Maximum Likelihood method based on the Tamura-Nei model (Tamura and Nei, 1993). Also, the evolutionary analysis was conducted using MEGA version 7.0 (Kumar et al., 2016).

### Analysis of microbial richness and diversity

Taxonomic abundance was presented in the phylum, class, order, family, and genus. Alpha diversity metrics were computed (observed species, Chao1, Shannon and inverse Simpson) among sponge samples using the QIIME package with a step size of 100 and 100 repetitions per step. These indices were presented to evaluate the richness and evenness of the associated bacteria within each sponge sample (Caporaso et al., 2010). To show whether the number of reads used in the analysis was sufficient in identifying species/OTU, rarefaction curves were calculated using the QIIME script alpha_rarefaction.py. (Caporaso et al., 2010). Good's coverage index was calculated as C = 1-(s/n), where “s” is the number of unique OTUs and “n” is the number of individuals in the sample (Naim et al., 2014). The beta diversity among the microbial communities in different sponges was evaluated using UniFrac analysis and the QIIME package (Caporaso et al., 2010). The phylogenetic tree was constructed with the FastTree program to cluster the samples by an unweighted-pair group method with arithmetic mean (UPGMA) using average linkages (Nam et al., 2011; Giles et al., 2013). Also, principal coordinate analysis (PCoA) plots were provided using the QIIME to visualize the effect of the microbial community on structuring the diversity in different sponges (Caporaso et al., 2010).

## Results

### Sponge taxonomic identification

In this study, 10 sponge samples were collected from three different sites in the Persian Gulf to evaluate their bacterial diversity (Table 1). Taxonomic identification of the sponges using a combination of multilocus DNA markers (28S rDNA and COI mtDNA) showed that the sponges belong to families Suberitidae, Pseudoceratinidae, Chondrillidae, Chalinidae, Halichondriidae, unclassified Dictyoceratida, and Irciniidae (Table 1).

### Bacterial richness and diversity analyses

A combined total of 134,495 raw pyrosequencing reads of the bacterial *16S rRNA* gene fragment comprising 52,835,851 bases were obtained from the sponge samples. Trimming and quality filtering of the raw reads derived 68,628 high-quality sequences. These denoised sequences were clustered at a 97% similarity into 724 unique OTUs. The highest number of OTUs was obtained from the sponge *Dictyoceratida* sp. (S11), representing 165 OTUs and minimum of 29 OTUs from the sponge *Suberites diversicolor* (S02).

In this study, 7.43% of *16S rRNA* gene fragments were unassigned OTUs at the phylum level. To have more detailed confirmation of the OTU representative sequences corresponding to unassigned, we extracted the sequences from each OTU and performed a BLAST search to check the sequences corresponding to the conserved region of the target region (*16S rDNA*). But the closest known sequences had less than 89% similarity rate (Supplementary Table 1) and the phylogenetic tree was not informative. Therefore, these OTUs were not included in subsequent analyses. The total number of reads retrieved and OTUs from each sponge are shown in Table 3.

**Table 3:** The observed number of operational taxonomic units (OTUs) and estimations of richness (Chao1) and diversity index (Shannon, Simpson) for 16S rRNA libraries of the Persian Gulf sponge samples.

**Sponge ID**	**Total reads**	**OTU richness**		**OTU diversity**		**Goods coverage (%)**
		**Observed OTUs**	**Chao1E**	**Shannon**	**Simpson**	
S02	2,886	29.0	34.25	2.58	0.75	99.7
S03	3,433	67.0	76.33	4.62	0.92	99.8
S04	5,769	49.0	52.0	4.03	0.92	99.9
S05	6,083	47.0	50.27	2.57	0.75	99.8
S06	11,423	75.0	80.14	2.40	0.67	99.9
S07	7,358	67.0	81.25	2.45	0.73	99.7
S09	6,552	55.0	58.33	4.46	0.93	99.9
S10	10,910	95.0	114.25	1.94	0.48	99.8
S11	7,584	165.0	165.0	4.77	0.87	99.9
S13	6,630	75.0	90.6	2.98	0.72	99.8

**Figure 1:**
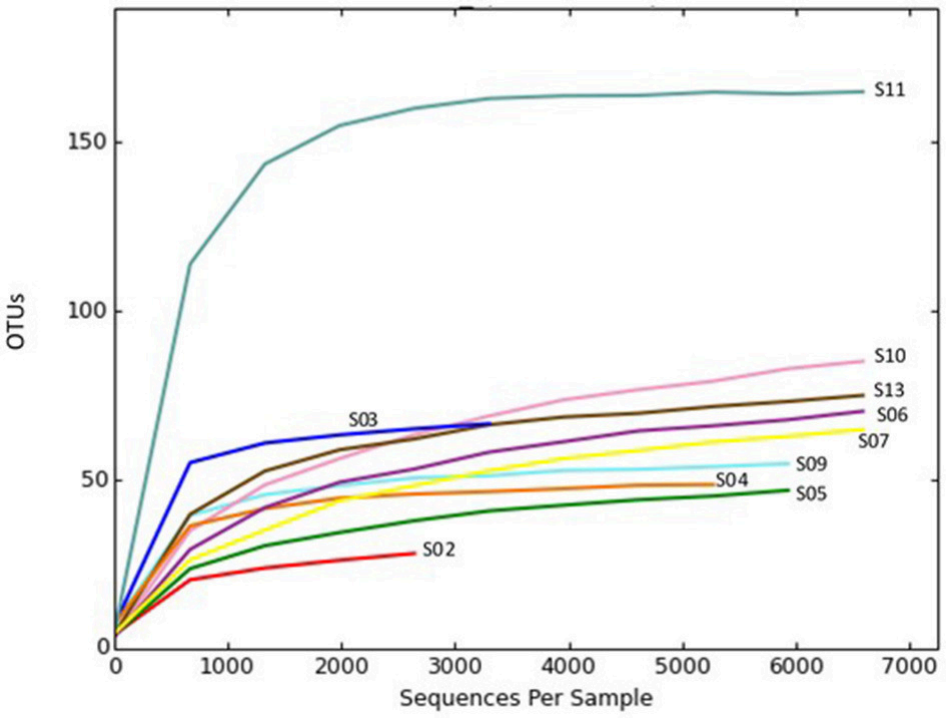
Rarefaction plot of OTU diversity in sponge samples collected from the Persian Gulf. Rarefaction curves were constructed at a 97% sequence similarity cut-off value by the QIIME package.

Rarefaction plots were constructed based on OTUs at a 97% sequence similarity cut-off value by the QIIME package. Alpha rarefaction curve showed that a reasonable number of reads have been used in analysis and identifying species/OTU. The sequencing depth of the sponge samples indicated the microbial communities were very well sampled (Figure 1). However, additional reads may be required to discover more OTUs for the samples such as S02 to show their total bacterial diversity. OTU based alpha diversity measures and Chao1 estimation of species richness revealed the highest richness of bacterial species in the sponge S11 and lowest in the sponge S02 (Table 3). Microbial community diversity, Shannon and Simpson indices displayed the highest community diversity and obviously distinguished the sponge S11 when compared to other sponge species collected from the same location (Table 3). In this study, mean and s.d. Expected richness (Chao1) was 80.242 ± 37.647, non-parametric Shannon (H′) was 3.28 ± 1.070 and Simpson (D) was 0.774 ± 0.141. Also, the obtained average of the Good's coverage index was 99.8 % ± 0.001 for all the sponge species.

### Taxonomic composition of bacterial pyrosequencing reads

**Figure 2:**
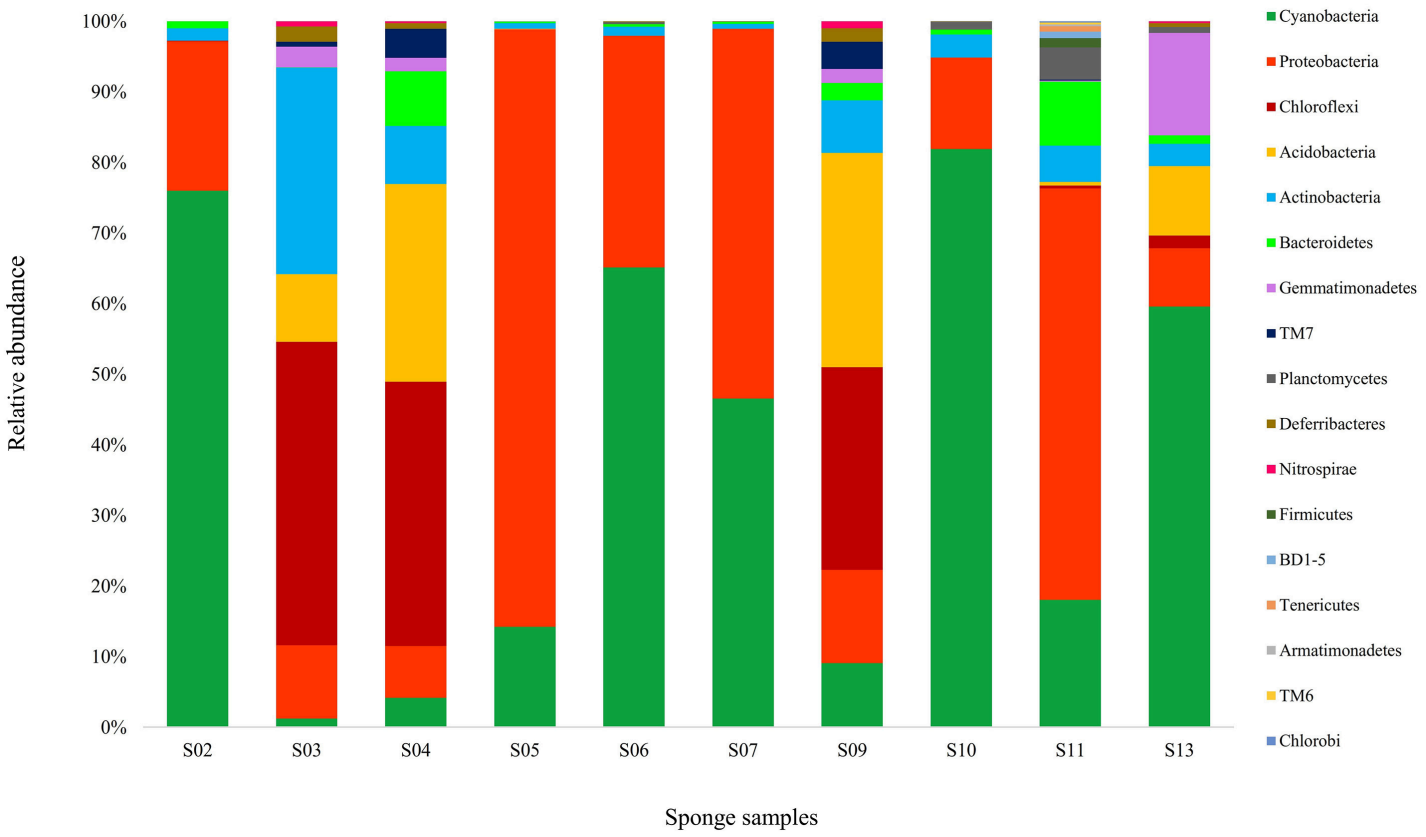
Taxonomic classification of bacterial sequences retrieved from different sponge samples collected from the Persian Gulf at the phylum level. Refer to Table 1 for sponge abbreviation.

Taxonomic assignment of the sequences of each OTU (68,628) was classified in the domains Bacteria (92.57% of the total dataset). Altogether, 17 bacterial phyla were recovered from the sponge samples. In the present study, 72.96% of the bacterial reads from the sponge samples were affiliated with two dominant phyla, Cyanobacteria (44.22%) and Proteobacteria (α-, β-, and γ- classes) (28.74%) (Figure 2). While the other reads belonging to Chloroflexi (8.67%), Acidobacteria (7.13%), Actinobacteria (4.72%), Bacteroidetes (2.04%), Gemmatimonadetes (2.04%), the candidate phylum TM7 (0.81%), Planctomycetes (0.69%), Deferribacteres (0.47%), Nitrospirae (0.19%), Firmicutes (0.10%), BD1-5 (0.07%), Tenericutes (0.06%), Armatimonadetes (0.02%), TM6 (0.02%), and Chlorobi (0.01%) were detected to be the minor groups in the sponge communities. Sponge S11 was obviously distinguished from the other sponges, in terms of containing taxa from 15 different bacterial phyla and candidate phyla. Other sponge samples contained 5 to 10 bacterial and candidate phyla. Also, the candidate phyla BD1-5 and TM6, and some phyla such as Firmicutes, Tenericutes, Armatimonadetes, and Chlorobi were only identified in sponge S11.

Diversity sponge-associated bacteria at the lower taxonomic levels showed that 45 classes and 87 orders were recovered from all datasets. *Cyanophyceae* (in Cyanobacteria), *Gammaproteobacteria, Alphaproteobacteria* were the major classes in the sponges, making up 43.78, 15.82, and 7.79%, respectively. The bacterial communities in sponges at the order level were heavily loaded with *Synechococcales* (43.78%) in Cyanobacteria and *Rhodobacterales* (16.32%) and *Rhizobiales* (9.42%) in Proteobacteria. At all the three levels of taxonomic classification, the bacterial community in the sponge S11 was more diverse than those associated with other sponges. Interestingly, sponge S11 has contained more rare bacteria compared with other 9 sponges. In addition, the bacterial compositions in the same sponges (e.g., S05 and S06) from similar sites did vary substantially (Figure 2).

### BLASTN and phylogenetic relationships of highly abundant OTUs

**Figure 3:**
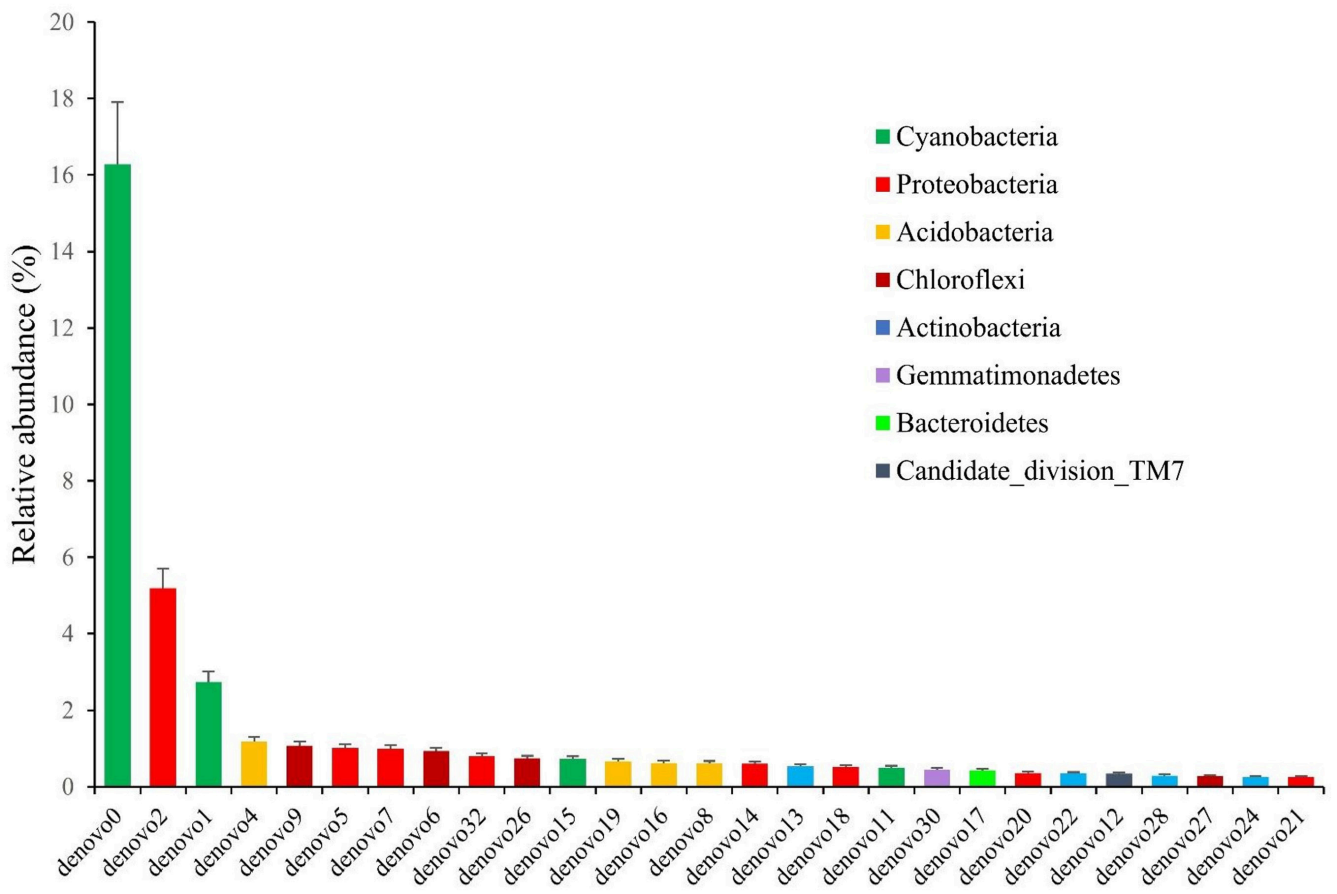
The relative abundance of OTUs with average proportions of more than 0.5%. The Standard Deviations (SD) were shown with error bars.

**Figure 4:**
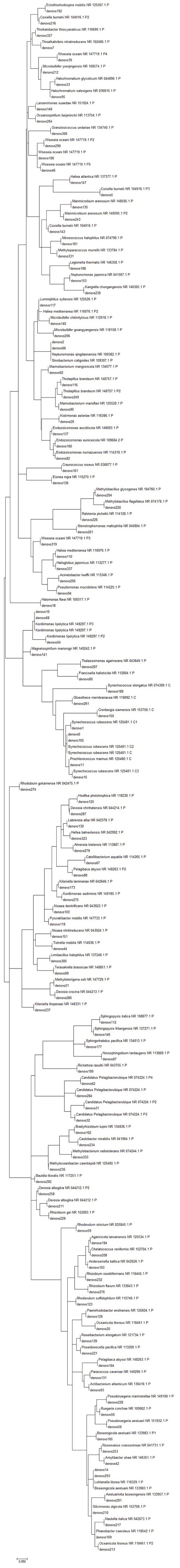
Molecular phylogenetic analysis of Cyanobacteria (red color) and proteobacteria (blue color) phyla in sponge samples based on the bacterial *16S rRNA* gene in GenBank. Tree topology constructed using Maximum Likelihood method, with bootstrap values >90%. Scale bar: 0.06 substitutions per nucleotide position.

To have more information about the sponge-associated bacteria strains, the representative sequences of each OTU were compared against the nucleotide database in GenBank. In this study, the first 27 OTUs with average proportions of more than 0.5% among sponge samples are shown in Figure 3. The most predominant OTU was OTU_denovo0 in the phylum Cyanobacteria, which accounted for proportions of 16.60% in the sponge-associated bacteria and shared the highest similarity (99%) to *Synechococcus rubescens* (Table 4). The second most dominant OTU was OTU_denovo2 in the phylum Proteobacteria, which accounted for proportions of 5.30% and shared 93% identity with the closest relative Proteobacteria bacterium *Sinobacterium caligoides* (Table 4). The phylum Proteobacteria was divided into more than 100 OTUs mainly belonging to *Gammaproteobacteria, Alphaproteobacteria*, and *Betaproteobacteria*. Following these symbionts in terms of relative abundance were OTU_ denovo1 in the phylum Cyanobacteria, OTU_denovo4 in Acidobacteria (read count: 1614), and OTU_denovo9 in Chloroflexi (read count: 1472) (Figure 3). Another highly abundant OUT in the phylum Cyanobacteria was related to OTU_denovo11 that affiliated with *Prochlorococcus marinus* with an identity of 97%. Details of all cyanobacterial and proteobacterial sequences included in the study are summarized in Table 4. Also, the evolutionary analysis of these two dominant phyla involved 127 nucleotide sequences and are shown in Figure 4.

**Table 4:** Details of all cyanobacterial and proteobacterial sequences included in the study.

**OTU**	**Read count**	**Phylum**	**Order**	**Family**	**Bacterial species**	**Strain**	**Accession no**.	**Similarity (%)**	**Habitat**	**Type source/place**
denovo0	22,341	Cyanobacteria	Synechococcales	Synechococcaceae	*Synechococcus rubescens*	SAG 3.81	NR_125481.1	99	Freshwater	Deep subalpine lakes (central Europe), lake Biwa (Japan), lake Balaton (Hungary), the Baltic Sea
denovo1	3,758	Cyanobacteria	Synechococcales	Synechococcaceae	*Synechococcus rubescens*	SAG 3.81	NR_125481.1	99	Freshwater	Deep subalpine lakes (central Europe), lake Biwa (Japan), lake Balaton (Hungary), the Baltic Sea
denovo11	686	Cyanobacteria	Synechococcales	Prochloraceae	*Prochlorococcus marinus*	PCC 9511	NR_125480.1	97	Seawater	North Atlantic, Mediterranean Sea
denovo15	993	Cyanobacteria	Synechococcales	Synechococcaceae	*Synechococcus rubescens*	SAG 3.81	NR_125481.1	99	Freshwater	Deep subalpine lakes (central Europe), Lake Biwa (Japan), Lake Balaton (Hungary), the Baltic Sea
denovo105	38	Cyanobacteria	Synechococcales	Synechococcaceae	*Synechococcus rubescens*	SAG 3.81	NR_125481.1	97	Freshwater	Deep subalpine lakes (central Europe), lake Biwa (Japan), lake Balaton (Hungary), the Baltic Sea
denovo150	35	Cyanobacteria	Nostocales	Nostocaceae	*Cronbergia siamensis*	SAG 11.82	NR_153750.1	96	NA	Germany
denovo189	19	Cyanobacteria	Synechococcales	Synechococcaceae	*Synechococcus elongatus*	PCC 6301	NR_074309.1	98	Freshwater	California
denovo261	12	Cyanobacteria	Chroococcales	Aphanothecaceae	*Gloeothece membranacea*	PCC 6501	NR_119092.1	96	NA	USA
denovo2	7,122	γ-Proteobacteria	Chromatiales	Chromatiaceae	*Nitrosococcus halophilus*	Nc 4	NR_074790.1	93	Marine	USA
denovo5	1,382	γ-Proteobacteria	Legionellales	Coxiellaceae	*Coxiella burnetii*	ATCC VR-615	NR_104916.1	92	NA	USA
denovo7	1,364	γ-Proteobacteria	Chromatiales	Ectothiorhodospiraceae	*Thioalkalivibrio nitratireducens*	DSM 14787	NR_102486.1	96	NA	Russia
denovo10	1,565	α-Proteobacteria	Kordiimonadales	Kordiimonadaceae	*Kordiimonas lipolytica*	M41	NR_149297.1	92	Seawater	China
denovo14	822	α-Proteobacteria	Rhodobacterales	Rhodobacteraceae	*Boseongicola aestuarii*	BS-W15	NR_133983.1	99	Sediment	Tidal flat sediment, South Korea
denovo18	696	γ-Proteobacteria	Oceanospirillales	Halomonadaceae	*Halomonas flava*	YIM 94343	NR_109317.1	93	Soil	Soil from salt lake, Qijiaojing Lake, China
denovo20	499	α-Proteobacteria	Rhodobacterales	Rhodobacteraceae	*Oceanicola litoreus*	M-M22	NR_118461.1	96	Sediment	Seashore sediment, Geoje island, South Korea
denovo21	340	α-Proteobacteria	Rhizobiales	Hyphomicrobiaceae	*Methyloterrigena soli*	M48	NR_147729.1	92	Soil	Activated sludge, Republic of Korea
denovo29	276	γ-Proteobacteria	Oceanospirillales	Hahellaceae	*Kistimonas asteriae*	KMD 001	NR_116386.1	95	NA	Republic of Korea
denovo31	274	α-Proteobacteria	Pelagibacterales	Pelagibacteraceae	*Candidatus Pelagibacterubique*	HTCC1062	NR_074224.1	99	Seawater	USA
denovo32	1086	α-Proteobacteria	Pelagibacterales	Pelagibacteraceae	*Candidatus Pelagibacterubique*	HTCC1062	NR_074224.1	100	Seawater	USA
denovo33	244	γ-Proteobacteria	Chromatiales	Chromatiaceae	*Halochromatium glycolicum*	BN 3201	NR_044896.1	95	NA	Germany
denovo39	105	γ-Proteobacteria	Chromatiales	Woeseiaceae	*Woeseia oceani*	XK5	NR_147719.1	93	Sediment	Coastal sediment, Xiaoshi Island, Weihai, China
denovo42	152	α-Proteobacteria	Rhodobacterales	Rhodobacteraceae	*Amylibacter ulvae*	KMM 6515	NR_146351.1	99	NA	Republic of Korea
denovo44	99	α-Proteobacteria	Rhodospirillales	Rhodospirillaceae	*Tistrella mobilis*	NBRC 102134	NR_114036.1	95	NA	Japan
denovo46	65	γ-Proteobacteria	Chromatiales	Woeseiaceae	*Woeseia oceani*	XK5	NR_147719.1	98	Sediment	Coastal sediment, Xiaoshi Island, Weihai, China
denovo50	142	α-Proteobacteria	Rhodobacterales	Rhodobacteraceae	*Pseudoruegeria aestuarii*	MME-001	NR_151932.1	96	Sediment	Tidal flat of Muui-do, Republic of Korea
denovo54	65	α-Proteobacteria	Kordiimonadales	Kordiimonadaceae	*Kordiimonas lipolytica*	M41	NR_149297.1	95	Seawater	China
denovo56	159	α-Proteobacteria	Rhodobacterales	Rhodobacteraceae	*Ruegeria conchae*	TW15	NR_109062.1	100	Ark clam	Strain: *Scapharca broughtonii*, South Korea
denovo59	79	α-Proteobacteria	Rhodobacterales	Rhodobacteraceae	*Rhodovulum strictum*	MB-G2	NR_025845.1	95	Seawater	Tidal and seawater pools, Japan
denovo62	73	α-Proteobacteria	Pelagibacterales	Pelagibacteraceae	*Candidatus Pelagibacterubique*	HTCC1062	NR_074224.1	94	Seawater	USA
denovo66	131	γ-Proteobacteria	Oceanospirillales	Oceanospirillaceae	*Neptunomonas qingdaonensis*	P10-2-4	NR_109382.1	94	Sand	Coastal area of Qingdao (Yellow Sea), China
denovo67	36	α-Proteobacteria	Rhodobacterales	Rhodobacteraceae	*Catellibacterium aquatile*	NBRC 104254	NR_114265.1	96	NA	Japan
denovo68	68	α-Proteobacteria	Kordiimonadales	Kordiimonadaceae	*Kordiimonas lipolytica*	M41	NR_149297.1	92	Seawater	China
denovo70	81	γ-Proteobacteria	Cellvibrionales	Halieaceae	*Haliea mediterranea*	7SM29	NR_116976.1	99	Seawater	Mediterranean sea water, Spain
denovo80	36	γ-Proteobacteria	Thiotrichales	Francisellaceae	*Francisella halioticida*	Shimane-1	NR_112804.1	99	Abalone	Strain: *Haliotis gigantean*, Japan
denovo82	41	γ-Proteobacteria	Oceanospirillales	Hahellaceae	*Endozoicomonas numazuensis*	HC50	NR_114318.1	99	Sponge	Strain: *Haliclona* sp., Coast of Numazu, Japan
denovo87	49	α-Proteobacteria	Sphingomonadales	Sphingomonadaceae	*Novosphingobium tardaugens*	NBRC 16725	NR_113869.1	99	NA	Japan
denovo90	31	γ-Proteobacteria	Oceanospirillales	Alteromonadaceae	*Marinobacterium marisflavi*	IMCC4074	NR_125520.1	93	Seawater	Atlantic Ocean, Yellow Sea, Incheon, Republic of Korea
denovo92	33	γ-Proteobacteria	Oceanospirillales	Alteromonadaceae	*Marinobacterium mangrovicola*	Gal22	NR_134077.1	97	Mangrove	Mangrove roots of *Rhizophora mangle*, Germany
denovo93	83	α-Proteobacteria	Rhodobacterales	Rhodobacteraceae	*Actibacterium atlanticum*	22II-S11-z10	NR_136418.1	96	NA	China
denovo94	56	γ-Proteobacteria	Pseudomonadales	Pseudomonadaceae	*Pseudomonas mucidolens*	NBRC 103159	NR_114225.1	100	NA	Japan
denovo95	35	γ-Proteobacteria	Chromatiales	Chromatiaceae	*Halochromatium salexigens*	6310: DSM 4395	NR_036810.1	94	Freshwater	Germany
denovo98	25	α-Proteobacteria	Rhodobacterales	Rhodobacteraceae	*Pelagibaca abyssi*	JLT2014	NR_148263.1	97	Seawater	China
denovo99	57	α-Proteobacteria	Rhizobiales	Methylocystaceae	*Terasakiella brassicae*	B3	NR_148851.1	93	Wastewater	Wastewater of a pickle-processing factory, China
denovo100	19	γ-Proteobacteria	Oceanospirillales	Hahellaceae	*Endozoicomonas euniceicola*	EF212	NR_109684.2	98	Octocoral	Strain: *Eunicea fusca* and *Plexaura* sp., coast of Florida, USA, and the coast of Bimini, Bahamas
denovo101	22	α-Proteobacteria	Rhodospirillales	Acetobacteraceae	*Craurococcus roseus*	NS130	NR_036877.1	92	Soil	Japan
denovo103	50	α-Proteobacteria	Rhodospirillales	Rhodospirillaceae	*Nisaea denitrificans*	DR41_21	NR_043923.1	95	Seawater	Coastal, surface waters of the north-western Mediterranean Sea, France
denovo104	32	α-Proteobacteria	Rhodobacterales	Rhodobacteraceae	*Pelagibaca abyssi*	JLT2014	NR_148263.1	95	Seawater	China
denovo110	35	γ-Proteobacteria	Cellvibrionales	Halieaceae	*Haliea mediterranea*	7SM29	NR_116976.1	98	Seawater	Mediterranean sea water, Spain
denovo112	15	α-Proteobacteria	Sphingomonadales	Sphingomonadaceae	*Sphingopyxis italica*	SC13E-S71	NR_108877.1	93	Soil	Tuff, volcanic rock of the Roman catacombs, Rome, Italy
denovo116	29	γ-Proteobacteria	–	–	*Thiolapillus brandeum*	Hiromi 1	NR_148757.1	95	Hydrothermal vent	Hydrothermal vent chimney, Okinawa, Japan
denovo117	62	γ-Proteobacteria	Cellvibrionales	Halieaceae	*Luminiphilus syltensis*	Ivo14	NR_125526.1	98	Sediment	Tidal flat, island of Sylt, North Sea, Germany
denovo119	19	α-Proteobacteria	Rhizobiales	Rhodobiaceae	*Pyruvatibacter mobilis*	GYP-11	NR_147733.1	95	Microalga	Marine microalga, Strain: *Picochloruma* sp., China
denovo123	37	α-Proteobacteria	Rhodobacterales	Rhodobacteraceae	*Rhodovulum sulfidophilum*	KC2142	NR_115746.1	92	Clinical sample	USA
denovo125	39	α-Proteobacteria	Rhizobiales	Phyllobacteriaceae	*Hoeflea phototrophica*	NCIMB 14078	NR_118230.1	96	Seawater	Yellow Sea, Republic of Korea
denovo126	14	α-Proteobacteria	Rhodobacterales	Rhodobacteraceae	*Paenirhodobacter enshiensis*	DW2-9	NR_125604.1	98	Soil	Sewage outlet of the Bafeng pharmaceutical factory, Enshi, Hubei province, PR China
denovo130	16	α-Proteobacteria	Rhodobacterales	Rhodobacteraceae	*Labrenzia alba*	5OM6	NR_042378.1	99	Oyster	Mediterranean coast, Spain
denovo131	60	α-Proteobacteria	Rhodobacterales	Rhodobacteraceae	*Paracoccus cavernae*	0511ARD5E5	NR_149299.1	95	Air	Ardales Cave,Malaga, Spain
denovo135	35	γ-Proteobacteria	Cellvibrionales	Halieaceae	*Marimicrobium arenosum*	CAU1038	NR_148595.1	94	Sea sand	Modo, Republic of Korea
denovo136	42	γ-Proteobacteria	Cellvibrionales	Cellvibrionaceae	*Eionea nigra*	17X/A02/237	NR_115270.1	92	Water	Waters of the coastal north-western Mediterranean Sea, France
denovo137	14	γ-Proteobacteria	Oceanospirillales	Hahellaceae	*Endozoicomonas ascidiicola*	AVMART05	NR_146693.1	95	Ascidian	*Ascidiella* sp., Gullmarsfjord, Sweden
denovo139	36	α-Proteobacteria	Rhodobacterales	Rhodobacteraceae	*Roseibacterium elongatum*	DFL-43	NR_121734.1	98	Sand	Australia
denovo140	20	γ-Proteobacteria	Cellvibrionales	Microbulbiferaceae	*Microbulbifer chitinilyticus*	ABABA 212	NR_112918.1	97	Mangrove	Mangrove forests, Okinawa, Japan
denovo141	20	α-Proteobacteria	Rhodospirillales	Rhodospirillaceae	*Magnetospirillum marisnigri*	SP-1	NR_149242.1	93	Freshwater	Sediments, three distinct locations in European Russia
denovo143	12	γ-Proteobacteria	Legionellales	Coxiellaceae	*Coxiella burnetii*	ATCC VR-615	NR_104916.1	97	NA	USA
denovo146	23	α-Proteobacteria	Sphingomonadales	Sphingomonadaceae	Sphingopyxis fribergensis	Kp5.2	NR_137271.1	94	Soil	Saxony, Germany
denovo147	15	γ-Proteobacteria	Cellvibrionales	Halieaceae	Haliea atlantica	SM1351	NR_137377.1	94	Seawater	Surface seawater of the Atlantic Ocean
denovo148	24	γ-Proteobacteria	Oceanospirillales	Halomonadaceae	*Larsenimonas suaedae*	ST307	NR_151924.1	95	Seepweed	Euhalophyte Suaeda salsa, Dongying, China
denovo151	18	α-Proteobacteria	Rhodospirillales	Rhodospirillaceae	*Nisaea nitritireducens*	DR41_18	NR_043924.1	94	Water	Coastal, surface waters of the north-western Mediterranean Sea, France
denovo153	13	γ-Proteobacteria	Oceanospirillales	Oceanospirillaceae	*Neptunomonas japonica*	JAMM 0745	NR_041567.1	95	Sediment	Sediment adjacent to sperm whale carcasses off Kagoshima, Japan
denovo162	22	α-Proteobacteria	Rhizobiales	Bradyrhizobiaceae	*Bradyrhizobium lupini*	USDA 3051	NR_134836.1	100	Nodule	Spain
denovo165	32	α-Proteobacteria	Rhodobacterales	Rhodobacteraceae	*Boseongicola aestuarii*	BS-W15	NR_133983.1	98	Sediment	Tidal flat sediment, South Korea
denovo169	24	α-Proteobacteria	Rhodobacterales	Rhodobacteraceae	*Phaeobacter caeruleus*	DSM 24564	NR_118542.1	99	Marine biofilm	Biofilm on stainless steel electrode, Genoa harbor, Italy
denovo173	10	α-Proteobacteria	Kiloniellales	Kiloniellaceae	*Kiloniella laminariae*	LD81	NR_042646.1	95	Marine macroalga	Strain: *Laminaria saccharina*,:Baltic Sea, Germany
denovo177	16	α-Proteobacteria	Sphingomonadales	Sphingomonadaceae	*Sphingorhabdus pacifica*	n34	NR_134813.1	99	Sediment	Sandy sediments of the Sea of Japan seashore
denovo181	16	γ-Proteobacteria	Chromatiales	Chromatiaceae	*Nitrosococcus halophilus*	Nc 4	NR_074790.1	93	Marine	USA
denovo186	11	γ-Proteobacteria	Chromatiales	Woeseiaceae	*Woeseia oceani*	XK5	NR_147719.1	98	Sediment	Coastal sediment, Xiaoshi Island, Weihai, China
denovo188	9	γ-Proteobacteria	Legionellales	Legionellaceae	*Legionella thermalis*	L-47	NR_146358.1	94	Water	Hot spring water, Tokyo, Japan
denovo192	16	γ-Proteobacteria	Chromatiales	Ectothiorhodospiraceae	*Ectothiorhodospira mobilis*	DSM 237	NR_125567.1	93	NA	NA
denovo193	9	α-Proteobacteria	Rhizobiales	Rhodobiaceae	*Anderseniella baltica*	BA141	NR_042626.1	98	Sediment	Surface of sediment in a deep basin of the central Baltic Sea, Germany
denovo194	37	α-Proteobacteria	Rhodobacterales	Rhodobacteraceae	*Agaricicola taiwanensis*	CC-SBABM117	NR_125534.1	96	Edible mushroom	Strain: *Agaricus blazei*, China
denovo199	10	α-Proteobacteria	Rickettsiales	Rickettsiaceae	*Rickettsia raoultii*	Khabarovsk	NR_043755.1	95	Tick	Strain: Dermacentor silvarum, Russia, France
denovo201	8	γ-Proteobacteria	Xanthomonadales	Xanthomonadaceae	*Stenotrophomonas maltophilia*	ATCC 19861	NR_040804.1	100	NA	Japan
denovo204	8	β-Proteobacteria	Nitrosomonadales	Methylophilaceae	*Methylobacillus glycogenes*	TK 0113	NR_104760.1	96	NA	NA
denovo206	8	γ-Proteobacteria	Cellvibrionales	Microbulbiferaceae	*Microbulbifer gwangyangensis*	GY2	NR_118158.1	98	Seawater	Tidal flat at Gwangyang Bay, Korea
denovo207	8	γ-Proteobacteria	Alteromonadales	Colwelliaceae	*Thalassomonas agarivorans*	TMA1	NR_043649.1	99	Water	Shallow coastal water of An-Ping Harbor, Taiwan
denovo208	14	α-Proteobacteria	Rhizobiales	Chelatococcaceae	*Chelatococcus reniformis*	B2974	NR_152704.1	96	Ice core	Muztagh Glacier, on the Tibetan Plateau, China
denovo210	15	α-Proteobacteria	Rhodobacterales	Rhodobacteraceae	*Silicimonas algicola*	KC90B	NR_152708.1	98	Diatom	Cell surface of the marine diatom, strain: *Thalassiosira delicatula*, France
denovo211	8	α-Proteobacteria	Rhizobiales	Hyphomicrobiaceae	*Devosia albogilva*	IPL15	NR_044212.1	99	Marine sediment	Yueqing Bay, Zhejiang Province, China
denovo212	28	γ-Proteobacteria	Cellvibrionales	Microbulbiferaceae	*Microbulbifer yueqingensis*	Y226	NR_108574.1	95	Dump	Hexachlorocyclohexane dump site, India
denovo213	7	α-Proteobacteria	Rhodobacterales	Rhodobacteraceae	*Oceanicola litoreus*	M-M22	NR_118461.1	99	Sediment	Seashore sediment, Geoje island, South Korea
denovo216	9	γ-Proteobacteria	Legionellales	Coxiellaceae	*Coxiella burnetii*	ATCC VR-615	NR_104916.1	97	NA	USA
denovo217	23	α-Proteobacteria	Rhodobacterales	Rhodobacteraceae	*Nautella italica*	LMG 24365	NR_042673.1	100	Marine biofilm	Marine electroactive biofilm, Genova harbor, Italy
denovo220	11	β-Proteobacteria	Nitrosomonadales	Methylophilaceae	*Methylobacillus flagellatus*	KT	NR_074178.1	96	NA	USA
denovo221	23	α-Proteobacteria	Rhodobacterales	Rhodobacteraceae	*Poseidonocella pacifica*	KMM 9010	NR_113209.1	99	Sediments	Shallow sandy sediments of the Sea of Japan
denovo226	29	β-Proteobacteria	Burkholderiales	Burkholderiaceae	*Ralstonia pickettii*	NBRC 102503	NR_114126.1	100	NA	Japan
denovo228	14	α-Proteobacteria	Rhodobacterales	Rhodobacteraceae	*Pseudoruegeria marinistellae*	SF-16	NR_149190.1	99	Starfish	Sanya, China
denovo229	7	α-Proteobacteria	Rhizobiales	Rhizobiaceae	*Rhizobium gei*	ZFJT-2	NR_152093.1	97	Plant	Stem of Geum aleppicum, Taibai Mountain, Shaanxi Province, north-west China
denovo230	16	γ-Proteobacteria	Oceanospirillales	Kangiellaceae	*Kangiella chungangensis*	CAU 1040	NR_148305.1	92	Marine sand	Jeju Island, South Korea
denovo232	7	α-Proteobacteria	Rhizobiales	Rhizobiaceae	*Rhizobium rosettiformans*	W3	NR_116445.1	100	Ground water	Lucknow, India
denovo234	14	α-Proteobacteria	Caulobacterales	Caulobacteraceae	*Caulobacter mirabilis*	FWC 38	NR_041964.1	97	Freshwater	Germany
denovo235	10	α-Proteobacteria	Rhizobiales	-	*Methyloceanibacter caenitepidi*	Gela4	NR_125465.1	99	Marine sediment	Near a hydrothermal vent, Japan
denovo237	14	α-Proteobacteria	Kiloniellales	Kiloniellaceae	*Kiloniella litopenaei*	P1-1	NR_148331.1	92	Soil	Hoh Xil basin, China
denovo243	8	γ-Proteobacteria	Cellvibrionales	Halieaceae	*Marimicrobium arenosum*	CAU1038	NR_148595.1	95	Sea sand	Modo, Republic of Korea
denovo249	11	γ-Proteobacteria	-	-	*Thiolapillus brandeum*	Hiromi 1	NR_148757.1	93	Hydrothermal vent	Hydrothermal vent chimney, Okinawa, Japan
denovo253	14	α-Proteobacteria	Rhodobacterales	Rhodobacteraceae	*Roseovarius crassostreae*	CV919-312	NR_041731.1	99	Oyster	Damariscotta River, USA
denovo258	10	α-Proteobacteria	Rhizobiales	Hyphomicrobiaceae	*Devosia albogilva*	IPL15	NR_044212.1	98	Marine sediment	Yueqing Bay, Zhejiang Province, China
denovo264	8	γ-Proteobacteria	Oceanospirillales	-	*Oceanospirillum beijerinckii*	NBRC 15445	NR_113754.1	95	NA	Japan
denovo274	11	α-Proteobacteria	Rhizobiales	Rhodobiaceae	*Rhodobium gokarnense*	JA173	NR_042475.1	95	Salt pan	Saltern Gokarna, India
denovo275	7	α-Proteobacteria	Kordiimonadales	Kordiimonadaceae	*Kordiimonas sediminis*	N39	NR_149185.1	94	Sediment	Sample collected at a sea cucumber culture pond in Weihai, China
denovo276	9	α-Proteobacteria	Rhizobiales	Rhizobiaceae	*Rhizobium flavum*	YW14	NR_133843.1	100	Soil	China
denovo279	8	α-Proteobacteria	Rhodobacterales	Rhodobacteraceae	*Ahrensia kielensis*	NBRC 15762	NR_113807.1	96	NA	Japan
denovo280	7	α-Proteobacteria	Rhizobiales	Hyphomicrobiaceae	*Devosia crocina*	IPL20	NR_044213.1	92	Dump	hexachlorocyclohexane dump site, India
denovo281	8	α-Proteobacteria	Rhodobacterales	Rhodobacteraceae	*Aestuariivita boseongensis*	BS-B2	NR_133957.1	98	Sediment	Tidal flat sediment, Boseong, South Korea
denovo284	18	α-Proteobacteria	Pelagibacterales	Pelagibacteraceae	*Candidatus Pelagibacterubique*	HTCC1062	NR_074224.1	95	Seawater	USA
denovo287	11	α-Proteobacteria	Rhizobiales	Hyphomicrobiaceae	*Devosia chinhatensis*	IPL18	NR_044214.1	95	Dump	Hexachlorocyclohexane (HCH) dump site, India
denovo290	7	γ-Proteobacteria	Chromatiales	Woeseiaceae	*Woeseia oceani*	XK5	NR_147719.1	97	Sediment	Coastal sediment, Xiaoshi Island, Weihai, China
denovo292	8	α-Proteobacteria	Rhizobiales	Hyphomicrobiaceae	*Bauldia litoralis*	524-16	NR_117251.1	93	NA	NA
denovo293	7	α-Proteobacteria	Rhodobacterales	Rhodobacteraceae	*Loktanella litorea*	DPG-5	NR_118329.1	100	Seawater	The South Sea, Republic of Korea
denovo295	8	γ-Proteobacteria	Pseudomonadales	Moraxellaceae	*Acinetobacter lwoffii*	JCM 6840	NR_113346.1	100	NA	Japan
denovo300	8	α-Proteobacteria	Rhodospirillales	Rhodospirillaceae	*Limibacillus halophilus*	CAU 1121	NR_137248.1	96	Soil	Reclaimed land in the Republic of Korea
denovo306	9	γ-Proteobacteria	Chromatiales	Granulosicoccaceae	*Granulosicoccus undariae*	W-BA3	NR_134740.1	94	Brown algae	Brown algae reservoir in Wando of South Korea
denovo319	9	γ-Proteobacteria	Chromatiales	Woeseiaceae	*Woeseia oceani*	XK5	NR_147719.1	95	Sediment	Coastal sediment, Xiaoshi Island, Weihai, China
denovo323	13	α-Proteobacteria	Rhodobacterales	Hyphomonadaceae	*Hellea balneolensis*	26III/A02/215	NR_042992.1	97	Freshwater	Surface water of the north-western Mediterranean Sea, France
denovo327	7	γ-Proteobacteria	–	–	*Thiohalobacter thiocyanaticus*	HRh1	NR_116699.1	93	Sediment	Mixed sediment from hypersaline chloride-sulfate lakes, Kulunda Steppe, Russia
denovo331	9	γ-Proteobacteria	Methylococcales	Methylococcaceae	*Methyloparacoccus murrellii*	R-49797	NR_133784.1	93	Pond water	South Africa
denovo333	7	α-Proteobacteria	Rhizobiales	Methylobacteriaceae	*Methylobacterium radiotolerans*	JCM 2831	NR_074244.1	100	NA	NA
denovo337	8	γ-Proteobacteria	Cellvibrionales	Halieaceae	*Halioglobus japonicus*	S1-36	NR_113277.1	100	Seawater	North-western Pacific Ocean near Japan

*OTU was determined based on a 16S rRNA gene similarity of 90%*.

*NA, Not available*.

### Clustering of sponges according to bacterial diversity

**Figure 5:**
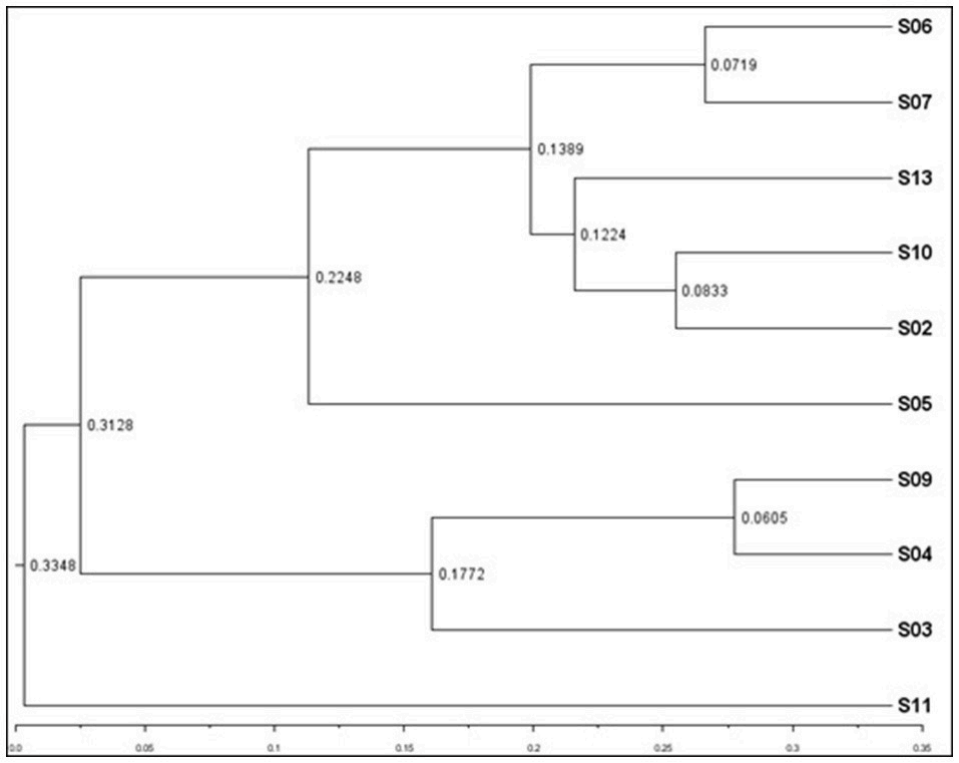
Weighted UPGMA tree from Unifrac analysis showing relationships between different sponges according to bacterial communities. The figure was constructed on the basis of tag pyrosequencing data. The scale bar represents the distance between clusters in UniFrac units.

**Figure 6:**
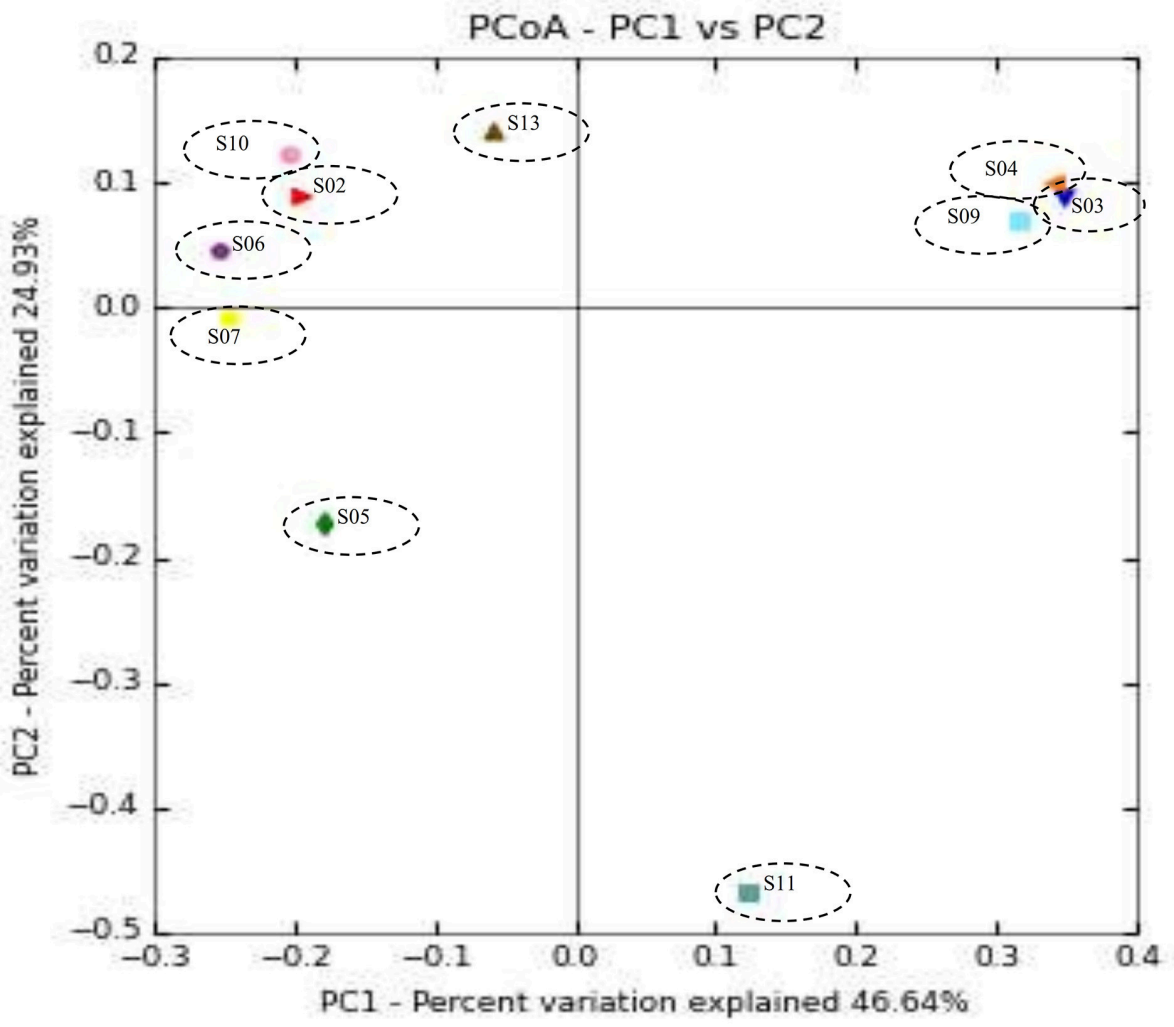
Principal coordinate analysis (PCoA) plot of sponge samples obtained with the unweighted UniFrac distance metric.

Weighted UPGMA tree from the Unifrac analysis was constructed to show the relationships between different sponges according to their bacterial communities (Figure 5). In this study, sponge S11 was always distinct from the others in all trees. Also, sponges S4 and S9 exhibited the same topology and the closest distance to each other. The association among other sponges was not noticeable in the Unifrac UPGMA tree topologies. Because the bacterial profiles in each sponge were highly different from each other. Further, the difference in the bacterial community structure of the sponge samples was evaluated using Principal coordinate analysis (PCoA) plot based on the unweighted unifrac distance metrics (Figure 6). Sponge S11 formed a distinct clade and showed notable differences from other sponges. Three pairs of sponges belonged to the same species (Table 1). But each pair was clustered in different clades except for the species *Chondrila* sp. (S04 and S09). Also, only the sponges S02, S10, and S13 contained Cyanobacteria as a major phylum.

## Discussion

Recently, marine sponges have been a major target of different studies due to their abundant and diverse microbial communities, ecological roles, production of novel bioactive natural compounds, and biotechnological significance (Menezes et al., 2010; Alex and Antunes, 2015). However, our understanding of bacteria-sponge interactions, nature, and diversity of bacteria associated with marine sponges is still incomplete. There are significant gaps in research on the bacterial composition, function, and maintenance of the symbiotic relationships (Menezes et al., 2010; Verhoeven et al., 2017). Deep sequencing approaches such as 454 tag pyrosequencing can be used to explore the microbial diversity of sponges with high efficiency rather than was possible by clone library construction and Sanger sequencing methods (Gao et al., 2014a; Moitinho-Silva et al., 2014).

### Taxonomic richness and bacterial community diversity

In this study, the number of unique OTUs (724) is in accordance with a study on 12 different marine sponge species from the Atlantic coast, where only 686 OTUs at 97% sequence similarity reported (Alex and Antunes, 2015). Here, the low diversity of OTUs in bacterial communities might be related to the bias of selected primers (V1–V3), in comparison to V3–V4 and V6 regions in other studies (Lee et al., 2011; Gaikwad et al., 2016; Souza et al., 2017). It will be led to inefficient amplification of the bacterial *16S rRNA* genes (Gao et al., 2017). On the other hand, differences in sampling depth and the water temperature may affect the number of OTUs in our sponge-associated bacterial communities than other studies (Jackson et al., 2012; Alex and Antunes, 2015; Souza et al., 2017). Another possibility may be because of the difference in using various bioinformatics patterns to analyze the sponge data. In CD-HIT-OTU program, when defining the OUT, there is a step to remove a cluster with fewer reads, such as a singleton or doubleton, as noise. This is not the same OTU picking method in MOTHUR or QIIME. It looks like the total number of OTU appears to be low when removing low size clusters. In the present study, the cutoff X value defined as the low size cluster was applied as 7, and the cluster consisting of less than 7 reads were removed without being picked by OTU. At this time, the total number of reads removed is 46,318.

In this study, alpha rarefaction curve showed that a reasonable number of reads have been used in analysis and identifying species/OTU. The bacterial richness estimation reported in the Persian Gulf sponges ranged from 29 to 165 OTUs. It is concordant with the range observed (29–370 OTUs) in a study targeting 12 marine sponge species sampled from the Atlantic coast (Alex and Antunes, 2015). In contrast, higher bacterial richness (570–3,013 OTUs) was found in the sponge-specific bacterial communities in Irish waters (Jackson et al., 2012) and also in the sponges from the Red Sea (251–444 OTUs) (Moitinho-Silva et al., 2014). Also, (Thomas et al., 2016) showed contributing marine sponges to the total bacterial diversity of the world's oceans, with a bacterial richness accounted for 50–3,820 OTUs in each sponge (Thomas et al., 2016). In the present study, diversity index and number of OTUs were higher in sponge S11 compared to other sponges. Sponge S11 was obviously distinguished from the other sponges, in terms of containing taxa from 15 different bacterial phyla and candidate phyla. At all the three levels of taxonomic classification, the bacterial community in the sponge S11 was more diverse than those associated with other studied sponges. Interestingly, sponge S11 has contained more rare bacteria compared with other sponges.

Taxonomic identification of sponge S11 using a combination of multilocus DNA markers showed that the sponge belongs to the genus of *Dictyoceratida* sp. Many studies have considered the order *Dictyoceratida* as the largest producer of new marine natural products, contributing more than 20% of all sponge-derived novel compounds (Mehbub et al., 2014, 2016). Different bacterial communities associated with the order *Dictyoceratida* are reported to produce a wide range of natural compounds with a variety of biological activities (Thakur et al., 2005; Mehbub et al., 2014, 2016). Also, Sponge S11 formed a distinct clade in the weighted UPGMA tree from Unifrac analysis and showed notable differences from other sponges.

Interestingly, in this study, the bacterial compositions in the same sponges (S05 and S06) from similar sites did vary substantially. There was a 100 m distance between the sampling sites of these two sponges. This finding confirms the previous studies, indicating that two cohabiting sponges may have different bacterial signatures (Jasmin et al., 2015; Jeong et al., 2015).

In the present study, a small number of OTUs (7.43%) were recorded as unassigned at the phylum level, after quality filtering and removal of chimera. When alignment is performed with UCLUST in the Reference DB, it means that there are no more than 90% references to match (OTU representative sequence). Unassigned OTUs may be a sequencing error element (Chimera, etc.) that could not be removed in the previous step (OTU picking step). Because there is no match result due to high cut off value, 90%. The frequency of unassigned OTUs was also maintained, after re-analysis of the OTU representative sequences corresponding to unassigned in a BLAST search for the conserved region of the target region (16S rDNA). These OTUs were not included in further analyses, but their presence is noteworthy. However, this number was much lower than those reported from Florida (White et al., 2012) and Indonesia (Cleary et al., 2013), where 36 and 34% of OTUs could not be assigned to any bacterial phylum, respectively.

### Community composition of sponge-associated bacteria

One of the highlights of our study was the high frequency of Cyanobacteria in the Persian Gulf sponges, contributing 44.22% of the total phylum-level diversity. Also, the presence of Cyanobacteria was confirmed in all the sponges studied with various abundance. Cyanobacteria were also the dominant phylum in the sponge samples reported from other tropical and subtropical regions (Alex et al., 2012; Gao et al., 2014a, 2017; Regueiras et al., 2017).

Within this phylum, our sponge samples contained a high proportion (97.37%) of free-living *Synechococcus*. BLAST search further revealed the dominant OTU in sponge samples and showed a high similarity with previously reported freshwater-specific species “*Synechococcus rubescens*” isolated from the deep subalpine lakes (central Europe), Lake Biwa (Japan), Lake Balaton (Hungary), and the Baltic Sea (Ernst et al., 2003). The Cyanobacteria *Synechococcus* has considered as an autotrophic plankton community and a substantial fraction of marine primary production (Flombaum et al., 2013), as sponge feeding on Cyanobacteria has been extensively confirmed (Pile et al., 1996; Hadas et al., 2009). This genus of marine bacteria has widely distributed in many ocean regions, covering both polar and high-nutrient waters (Flombaum et al., 2013). Furthermore, the cyanobacterial *Synechococcus* lineage is believed to have several ecotypes that are adapted to different environmental conditions including light, temperature, nutrients, and chlorophyll a concentration (Flombaum et al., 2013). It is possible that the core OTUs illustrate bacterial sponge ecotypes that are matched to the niche sponges and are probably environmentally transmitted (Schmitt et al., 2012). As Cyanobacteria are the center of carbon fixation and provide necessary nutrients to photosynthetic sponge hosts (Taylor et al., 2007), the high abundance of Cyanobacteria further indicated the specific roles of photosynthetic bacteria and their profitability in sponge biology (Lemloh et al., 2009; Alex et al., 2012).

The Persian Gulf sponges were recurrently exposed to light in low depth (< 3 m) in this study. Therefore, the prominence of photosymbionts was predictable in these sponges. In spite of the important role of Cyanobacteria, they are typically absent in Antarctic sponges (Rodríguez-Marconi et al., 2015). It seems that environmental factors such as temperature, salinity or nutrient levels might impact the composition of bacterial community structures in different sponges (Giles et al., 2013; Cuvelier et al., 2014).

The co-evolution and functional aspects of sponge-cyanobacteria associations have not been revealed in details. Sponges may acquire their cyanobacterial symbionts by vertical, horizontal or combined transmission routes (Thacker and Freeman, 2012). The genome-level research on cyanobacterial symbionts of sponges showed that they have general and specific adaptations to life within the sponge host in comparison with free-living cyanobacteria (Gao et al., 2014b; Burgsdorf et al., 2015). Sponge symbionts have adapted mechanisms to actively seek out by their sponge hosts (Webster and Thomas, 2016). For example, cyanobacterial symbionts contains large number eukaryotic-like domains, such as ARs. These domains may be involved in avoiding digestion by the sponge host (Gao et al., 2017).

Lifestyle evolutionary and functional studies on other functions enriched and depleted in cyanobacterial symbionts of sponges compared to members of the closely related free-living strains reveled the precise and smart adaptation of cyanobacteria to live in full of challenge sponge's intercellular environment. Sponge amoebocytes may not actively distinguish between food bacteria and their cyanobacterial symbionts (Webster and Thomas, 2016). Hence, the depleted genes involved in biosynthesis of LPS O antigen in cyanobacterial symbionts of sponges produces a defense mechanism against sponge predation and phage attack (Burgsdorf et al., 2015; Webster and Thomas, 2016). In functional profile studies of bacterial symbionts with sponges, common functions in similar niches were found, indicating functionally convergence of symbionts in the divergent hosts (Fan et al., 2012; Rua et al., 2015). The biological and ecological roles of these functional equivalences may be of general importance for the adaptation of cyanobacterial symbiont to the sponge host environment and other symbiotic interactions.

In the present study, Proteobacteria was the second most abundant phylum in the microbiome of sponge-associated bacteria. Our study is in accordance with other studies showing proteobacteria as one of the most diverse phyla of sponge-isolated bacterial communities, irrespective of the habitat (Menezes et al., 2010; Schmitt et al., 2012; Jeong et al., 2014, 2015; Gao et al., 2017). In Proteobacteria phylum, OTUs were mainly affiliated with *Gammaproteobacteria, Alphaproteobacteria, Betaproteobacteria* classes, respectively. Multiple studies have highlighted the presence of *Gammaproteobacteria* in marine invertebrates, such as sponges (Menezes et al., 2010; Giles et al., 2013; Graça et al., 2015; Rodríguez-Marconi et al., 2015), corals (Sun et al., 2014), and oysters (Garnier et al., 2007). In this study, *Gammaproteobacteria* accounted for 15.82% of the total bacterial community, including mainly isolates belonging to genus *Nitrosococcus*. Different species of the genus *Nitrosococcus* are well known as ammonia-oxidizing bacteria (AOB), inducing the process of nitrification in different sponges and removing the ammonia excreted by the sponge host (Hentschel et al., 2006; Gao et al., 2017). The combined action between AOB and nitrite-oxidizing bacteria (NOB), such as members of the phylum Nitrospirae might then be responsible for the conversion of ammonia to nitrate in sponges (Bayer et al., 2007; Han et al., 2013). In the present study, nitrite-oxidizing phylum Nitrospirae (0.19%) was identified as one of the minor phyla in the Persian Gulf sponges. Our study is in accordance to other pyrosequencing studies in which Nitrospirae constituted a small portion of reads, accounted for 0.01–3% among several sponge species (Webster et al., 2010; Lee et al., 2011; Bayer et al., 2014; Gaikwad et al., 2016).

Chloroflexi was ranked as the third most abundant group (8.67%) using our sequencing approach. In this study, sponge 04 (*Chondrilla* sp.) harbored the largest proportion of Chloroflexi in comparison to other sponges. The presence of this phylum was previously reported in the Mediterranean sponge *Chondrilla nucula* using a clone library of *16S rRNA* gene sequences (Thiel et al., 2007). The Chloroflexi is one of the most common and diverse bacterial phyla associated with a wide range of sponges, with many sponge-specific lineages detected (Schmitt et al., 2011; Hardoim et al., 2012; Jeong et al., 2013; Gao et al., 2017). Different studies have revealed the important role of Chloroflexi in nutrition, defense (Hardoim et al., 2012) and carbon fixation through photosynthesis in marine sponges (Brück et al., 2010), indicating its autotrophic lifestyle. In the present study, the Chloroflexi OTUs were closer to the autotrophic lineages.

Actinobacteria was another phylum inhabiting the Persian Gulf sponges. This phylum has been widely reported in marine sponges (Schmitt et al., 2012; Giles et al., 2013; Bayer et al., 2014; Cuvelier et al., 2014; Naim et al., 2014). Different studies have been considered Actinobacteria as an important source of bioactive natural products (Izumikawa et al., 2010; Pimentel-Elardo et al., 2010; Abdelmohsen et al., 2012), protecting the sponge hosts against pathogens (O'Connor-Sánchez et al., 2014). There is a possibility that this phylum may provide new opportunities for novel marine drug discovery.

The candidate division TM7, also known as phylum candidatus Saccharibacteria, is a highly ubiquitous and uncultured phylum of bacteria, described through environmental *16S rRNA* gene sequence and genome data only (Ferrari et al., 2014). The existence of this phylum has widely been reported from different sponges (Webster et al., 2010; Lee et al., 2011; Schmitt et al., 2012; Gao et al., 2014a; Montalvo et al., 2014; Alex and Antunes, 2015; Gaikwad et al., 2016). In the present study, TM7 was found at very low abundance in some sponge species. It highlights the importance of deep sequencing technology for detection of the sponge-associated uncultivated bacteria and rare microbial groups in sponges. Otherwise, they might have been not discovered by other routine molecular methods (Lee et al., 2011). However, because of the lack of cultivated representatives and minimal genomic sampling knowledge on the metabolism and biological activities of this enigmatic group has been remained unclear (Ferrari et al., 2014).

The candidate phylum Poribacteria is a sponge-specific phylum that has been widely detected and described in a variety of sponge species (Lafi et al., 2009; Cleary et al., 2013). This candidate phylum has typically been reported in high microbial abundance (HMA) sponge microbiomes (Hochmuth et al., 2010) and considered as “indicator species” for these group of sponges (Bayer et al., 2014). However, unexpectedly, no Poribacteria was found in sponges of the present study.

One reason for the lack of Poribacteria in the Persian Gulf sponges could be related to the use of different primers and bioinformatics pipelines (Souza et al., 2017). Also, some studies have shown that Poribacteria has not been associated with the orders such as *Halichondrida, Dictyoceratida*, and *Haplosclerida* (Lafi et al., 2009; Jeong et al., 2013). Noteworthy, these orders have been among the six orders found in this study. In addition, in contrast to other studies conducted on sponges (Kennedy et al., 2008; Montalvo et al., 2014; Alex and Antunes, 2015), the Verrucomicrobia and Spirochaetes phyla were not observed in the bacterial community of the Persian Gulf sponges.

Our study showed that more than 90% of the microbial groups observed in the Persian Gulf sponges were also represented in other studies conducted on the seawater samples (Lee et al., 2011; Schmitt et al., 2011; Gao et al., 2014a; Alex and Antunes, 2015). Nitrospirae, Tenericutes, Armatimonadetes, and the two candidate phyla BD1-5 and TM6 were the only bacterial communities reported exclusively in association with the marine sponges.

Our results showed that the representative OTUs sequences were related to sequences mainly from marine environments such as seawater and marine sediments. Interestingly, sponges had a very low proportion of the isolation source in the closest relative bacteria. Also, some sequences were related to other marine invertebrates such as abalone, octocoral, sea cucumber, oyster, ascidian, starfish, etc. This finding supports the hypothesis of possible environmental acquisition and/or horizontal transmission of bacteria (Moitinho-Silva et al., 2014; Alex and Antunes, 2015). It seems that innate immune system in sponges is responsible for differentiation between symbionts and food microbes (Müller and Müller, 2003). As a result, some overlap between the bacteria in the surrounding seawater and marine sponges would occur. A further deep-sequencing approach is needed to improve our knowledge about the nature of the bacterial specificity among the marine sponges (Alex and Antunes, 2015).

## Conclusion

In this study, evaluation of 16S rRNA gene amplicon tag pyrosequencing data showed a complex structure of the previously uncharacterized bacterial communities associated with the Persian Gulf sponges (class *Demospongiae*). OTU-based description of the bacterial communities exhibited altogether 17 different bacterial phyla, containing 14 formally described phyla and three candidate phyla. The dominance of Cyanobacteria may suggest an ecological importance of this phylum in the Persian Gulf sponges. More specifically, most of the bacterial symbionts were previously described as significant participants in the carbon, nitrogen cycle, and chemical defense of the studied sponges. Sponge S11 was highly diverse in comparison to other studied sponges and contained more rare bacteria. More research is needed to fully understand the composition of the Persian Gulf sponge-associated prokaryotic communities, specifically the functionality of these specific microbiomes as an important part of the marine ecosystem.

## Author contributions

IN and AN designed the research. AN and MM performed the experiments. AN analyzed the data. AN and IN wrote the manuscript with contributions from all authors.

### Conflict of interest statement

The authors declare that the research was conducted in the absence of any commercial or financial relationships that could be construed as a potential conflict of interest.
