# Supplementary material for: First Record of Microbiomes of Sponges Collected From the Persian Gulf, Using Tag Pyrosequencing
Source: Front Microbiol. 2018 Jul 6;9:1500. doi: 10.3389/fmicb.2018.01500 (PMC6043863; doi:10.3389/fmicb.2018.01500)
Supplement: Supplementary file 1 [file Table_1.DOCX]

Supplementary Table 1. Unassigned OTUs in the sponge samples

| **OUT No.** | **Closest BLAST relative** | **Accession No.** | **% similarity** |
| --- | --- | --- | --- |
| 10 | Proteobacteria; Kordiimonadaceae; Kordiimonas lipolytica strain M41  Proteobacteria; Kordiimonadaceae; Kordiimonas.gwangyangensis strain GW14-5  Proteobacteria; Kordiimonadaceae; Kordiimonas aestuarii strain 101-1 | NR_149297.1  NR_043103.1  NR_109376.1 | 87% |
| 43 | Bacteroidetes; Rhodothermaceae; Rhodothermus marinus strain DSM 4252  Bacteroidetes; Rhodothermaceae; Rhodothermus marinus strain R-10  Bacteroidetes; Rhodothermaceae; Rhodothermus marinus strain DSM 4252 | NR_074728.1  NR_029282.2  NR_114548.1 | 84% |
| 53 | Actinobacteria; Motilibacteraceae; Motilibacter peucedani strain RP-AC37  Actinobacteria; Motilibacteraceae; Motilibacter rhizosphaerae strain RS-16 | NR_108466.1  NR_108467.1 | 78% |
| 64 | Proteobacteria; Bdellovibrionaceae; Bdellovibrio exovorus strain JSS  Proteobacteria; Bdellovibrionaceae; Bdellovibrio exovorus strain JSS | NR_102876.1  NR_116108.1 | 82% |
| 68 | Proteobacteria; Kordiimonadaceae; Kordiimonas lipolytica strain M41  Proteobacteria; Caulobacteraceae; Asticcacaulis. excentricus strain CB 48  Proteobacteria; Caulobacteraceae; Asticcacaulis. excentricus strain CB 48 | NR_149297.1  NR_074137.1  NR_114730.1 | 87% |
| 75 | Proteobacteria; Alphaproteobacteria; Rickettsiales; Lyticum flagellatum strain 299  Proteobacteria; Alphaproteobacteria; Rickettsiales; Occidentia massiliensis strain Os18 | NR_125566.1  NR_149220.1 | 80% |
| 77 | Actinobacteria; Pseudonocardiaceae; Lentzea albidocapillata strain NBRC 100372  Actinobacteria; Pseudonocardiaceae; Lentzea albidocapillata strain IMMIB D-958 | NR_112664.1  NR_119177.1 | 80% |
| 84 | Tenericutes;; Mycoplasmataceae; Mycoplasma iowae strain 695  Tenericutes; Spiroplasmataceae; Spiroplasma clarkii strain CN-5 | NR_044669.2  NR_104750.1 | 79% |
| 107 | Actinobacteria; Pseudonocardiaceae; Allokutzneria multivorans strain YIM 120521  Actinobacteria; Pseudonocardiaceae; Kutzneria chonburiensis strain SMC 256  Actinobacteria; Pseudonocardiaceae;Labedaea rhizosphaerae strain RS-49 | NR_109545.1  NR_145619.1  NR_108468.1 | 79% |
| 115 | Proteobacteria; Chromatiaceae; Marichromatium bheemlicum strain JA124  Proteobacteria; Chromatiaceae; Thiorhodococcus drewsii strain AZ1 | NR_042477.1  NR_116895.1 | 83% |
| 128 | Cyanobacteria; Coleofasciculaceae; Coleofasciculus chthonoplastes strain SAG 2209  Cyanobacteria; Oscillatoriaceae; Oscillatoria sancta strain PCC 7515 | NR_125521.1  NR_112112.1 | 79% |
| 155 | Tenericutes; Mollicutes; Mycoplasmataceae; Ureaplasma diversum strain A417  Tenericutes; Mollicutes; Mycoplasmataceae; Ureaplasma cati strain F2  Tenericutes; Mollicutes; Mycoplasmataceae; Ureaplasma canigenitalium strain D6P-C | NR_025878.1  NR_115604.1  NR_025877.1 | 78% |
| 187 | Verrucomicrobia; Rubritaleaceae; Rubritalea halochordaticola strain MN1-1006  Verrucomicrobia; Rubritaleaceae; Rubritalea sabuli strain YM29-052 | NR_113049.1  NR_041630.1 | 78% |
| 196 | Actinobacteria; Sporichthyaceae; Sporichthya polymorpha strain DSM 43042  Actinobacteria; Kineosporiaceae; Kineococcus glutinatus strain YIM 75677 | NR_024727.1  NR_109547.1 | 79% |
| 218 | Verrucomicrobia; Puniceicoccaceae; Coraliomargarita akajimensis strain DSM 45221  Verrucomicrobia; Puniceicoccaceae; Coraliomargarita akajimensis strain 04OKA010-24  Verrucomicrobia; Verrucomicrobiaceae; Fucophilus fucoidanolyticus strain SI-1234 | NR_074901.1  NR_041496.1  NR_112167.1 | 85% |
| 224 | Acidobacteria; Pyrinomonadaceae; Brevitalea deliciosa strain Ac_16_C4  Acidobacteria; Pyrinomonadaceae; Brevitalea aridisoli strain Ac_11_E3 | NR_151988.1  NR_151987.1 | 79% |
| 236 | Proteobacteria; Thiotrichaceae; Thiothrix unzii strain A1  Proteobacteria; Thiotrichaceae; Thiothrix fructosivorans strain Q | NR_118100.1  NR_104845.1 | 83% |
| 238 | Proteobacteria; Rhodospirillaceae; Constrictibacter antarcticus strain 262-8  Chloroflexi; Caldilineaceae; Caldilinea tarbellica strain D1-25-10-4 | NR_112948.1  NR_117797.1 | 76% |
| 245 | Cyanobacteria; Oscillatoriophycideae; Oscillatoriales; unclassified Oscillatoriales; Potamolinea aerugineo-caerulea strain 1PC | NR_151861.1 | 89% |
| 248 | Proteobacteria; Methylococcaceae; Methylobacter marinus strain A45  Proteobacteria; Methylococcaceae; Methylobacter luteus strain NCIMB 11914 | NR_025132.1  NR_041814.1 | 83% |
| 266 | Proteobacteria; Rhodospirillaceae; Constrictibacter antarcticus strain 262-8  Proteobacteria; Acetobacteraceae; Craurococcus roseus strain NS130 | NR_112948.1  NR_036877.1 | 82% |
| 267 | Proteobacteria; Halobacteriovoraceae; Bacteriovorax litoralis strain JS5  Proteobacteria; Halobacteriovoraceae; Halobacteriovorax marinus strain SJ  Proteobacteria; Halobacteriovoraceae; Bacteriovorax marinus SJ strain SJ | NR_028724.1  NR_102485.1  NR_028723.1 | 80% |
| 268 | Proteobacteria; Chromatiaceae; Candidatus Thiodictyon syntrophicum strain Cad16  Proteobacteria; Chromatiaceae; Thiodictyon elegans strain DSM 232 | NR_114886.1  NR_044363.1 | 82% |
| 270 | Proteobacteria; Sphingomonadaceae; Sphingomonas xinjiangensis strain 10-1-84  Proteobacteria; Oceanospirillales; Marinomonas gallaica  Proteobacteria; Hyphomicrobiaceae; Paradevosia shaoguanensis strain J5-3 | NR_108386.1  NR_151935.1  NR_136441.1 | 88% |
| 282 | Proteobacteria; Oceanospirillales; Marinomonas gallaica  Proteobacteria; Oceanospirillales; Marinobacterium marisflavi strain IMCC4074 | NR_151935.1  NR_125520.1 | 87% |
| 298 | Spirochaetes; Spirochaetaceae; Salinispira pacifica strain L21-RPul-D2  Spirochaetes; Spirochaetaceae; Spirochaeta aurantia strain J1 | NR_134803.1  NR_104782.1 | 83%  80% |
| 299 | Spirochaetes; Spirochaetaceae; Salinispira pacifica strain L21-RPul-D2  Spirochaetes; Spirochaetaceae; Spirochaeta isovalerica strain DSM 2461 | NR_134803.1  NR_117137.1 | 83%  815 |
| 302 | Actinobacteria; Mycobacteriaceae; Mycobacterium saskatchewanense strain NRCM  Actinobacteria; Mycobacteriaceae; Mycobacterium nebraskense strain ATCC BAA-837 | NR_042793.1  NR_117224.1 | 75%  75% |
| 304 | Actinobacteria; Streptomycetaceae; Streptomyces vastus strain NRRL B-12232  Actinobacteria; Streptomycetaceae; Streptomyces vastus strain NBRC 13094 | NR_043841.1  NR_112366.1 | 76%  76% |
| 308 | Proteobacteria; Desulfobacteraceae; Desulforegula conservatrix strain Mb1Pa  Proteobacteria; Desulfohalobiaceae; Desulfonauticus autotrophicus strain DSM 4206  Proteobacteria; Desulfovibrionaceae; Desulfovibrio capillatus strain Met 2 | NR_028780.1  NR_044591.1  NR_115158.1 | 77% |
| 315 | Proteobacteria; Hydrogenophilaceae; Tepidiphilus margaritifer strain N2-214 | NR_025556.1 | 77% |
| 318 | Proteobacteria; Rhodobiaceae; Rhodobium gokarnense strain JA173  Proteobacteria; Rhodobiaceae; Rhodobium orientis strain MB312 | NR_042475.1  NR_029128.1 | 84% |
| 320 | Actinobacteria; Microbacteriaceae; Agromyces ulmi strain XIL01 | NR_029108.1 | 72% |
| 321 | Chloroflexi; Anaerolineaceae; Anaerolinea thermophila strain UNI-1  Chloroflexi; Anaerolineaceae; Anaerolinea thermophila strain UNI-1 | NR_074383.1  NR_036818.1 | 71%  72% |
| 322 | Bacteroidetes; Flammeovirgaceae; Flexithrix dorotheae strain NBRC 15987  Bacteroidetes; Flammeovirgaceae; Flexithrix dorotheae strain ATCC 23163  Bacteroidetes; Flammeovirgaceae; Flexithrix dorotheae strain IFO 15987 | NR_113831.1  NR_112649.1  NR_040919.1 | 83% |
| 325 | Spirochaetes; Spirochaetaceae; Sphaerochaeta pleomorpha strain Grapes  Spirochaetes; Spirochaetaceae; Sphaerochaeta pleomorpha strain Grapes | NR_102964.1  NR_074101.1 | 77% |
| 326 | Proteobacteria; Kangiellaceae; Kangiella profundi strain FT102  Proteobacteria; Kangiellaceae; Kangiella koreensis strain DSM 16069  Proteobacteria; Kangiellaceae; Kangiella koreensis strain SW-125 | NR_136491.1  NR_074715.1  NR_027574.1 | 83% |
| 329 | Deinococcus-Thermus; Trueperaceae; Truepera radiovictrix strain RQ-24  Deinococcus-Thermus; Trueperaceae; Truepera radiovictrix strain RQ-24 | NR_074381.1  NR_043482.1 | 87% |
| 332 | Proteobacteria; Anaplasmataceae; Ehrlichia chaffeensis strain Arkansas  Proteobacteria; Anaplasmataceae; Ehrlichia ruminantium strain Welgevonden  Proteobacteria; Anaplasmataceae; Ehrlichia minasensis strain UFMG-EV | NR_074500.2  NR_074513.2  NR_148800.1 | 85% |
| 336 | Spirochaetes; Spirochaetaceae; Spirochaeta cellobiosiphila strain SIP1  Spirochaetes; Spirochaetaceae; Spirochaeta psychrophila strain MO-SPC1 | NR_044505.1  NR_134185.1 | 87%  84% |
